# Renal carcinoma infiltrating inferior vena cava and combined valvular heart disease - one-stage uro-cardiological procedure: a case report

**DOI:** 10.1186/1477-7819-8-63

**Published:** 2010-07-28

**Authors:** Artur A Antoniewicz, Slawomir Poletajew, Andrzej Biederman, Lukasz Zapala, Andrzej Borowka

**Affiliations:** 1 Department of Urology, The Medical Centre of Postgraduate Education, Warsaw, Poland; 2 First Department of Cardiac Surgery, Institute of Cardiology, Warsaw, Poland

## Abstract

Standard treatment of patients with coexisting cardiac and non-cardiac diseases includes two separate operations. We report a case of 55-year-old man with combined valvular heart disease and renal carcinoma infiltrating inferior caval vein, who underwent one-stage cardio-urologic procedure. In the first step, mitral and tricuspid valvuloplasty were performed by cardiac surgeons. Then, urologists performed radical nephrectomy and thrombectomy. The postoperative course was uneventful. In twelve months follow-up the patient shows no signs of reccurrence and he had no symptoms of cardiac disease. To the best of our knowledge such a case has never been reported before in the literature.

## Background

Coexistence of cardiac and non-cardiac diseases requiring surgical treatment has been a matter of debate for many years. The major problem concerns patients suffering from cardiac and oncologic diseases. The strategy of two separate procedures should be taken into consideration when consulting such a case. However, if the cardiac operation is performed first, the oncologic treatment is delayed and the chances for success are poorer. Furthermore, the immunosuppressive effect of extracorporeal circulation may accelerate tumor growth and disseminate cancer cells [1]. If oncologic operation is performed first, the risk of operation is very high due to heart status. There is also an aspect of risk and cost of two hospital stays and additional anaesthesia.

In this group of patients, cardiac and non-cardiac operation performed under single anaesthesia seems to be interesting therapeutic option. However, the combined procedure requires thorough operation plan and two experienced, harmonious surgical teams. Some surgeons have started to perform such procedures. Satisfactory results are reported concerning one-stage cardiac operation and pulmonary tumor resection [2], carotid endarterectomy [3], abdominal aortic aneurysm repair [4], resection of goiter [5] and others.

Till now there has been just few publications on one-stage cardio-urologic operations [6-9] and there are no reports concerning patients with combined valvular heart disease and urologic tumor.

## Case presentation

55-year-old man (height 1,78 m; weight 70 kg) with severe heart failure - NYHA class III/IV was admitted to cardiology department for evaluation for surgery of incompetent mitral and tricuspid valves. Transthoracic echocardiogram confirmed diagnosis of severe mitral and tricuspid incompetence, dilated left ventricle, poor contractility (EF - 40%), pulmonary hypertension (PASP 90 mmHg).

**Figure 1:**
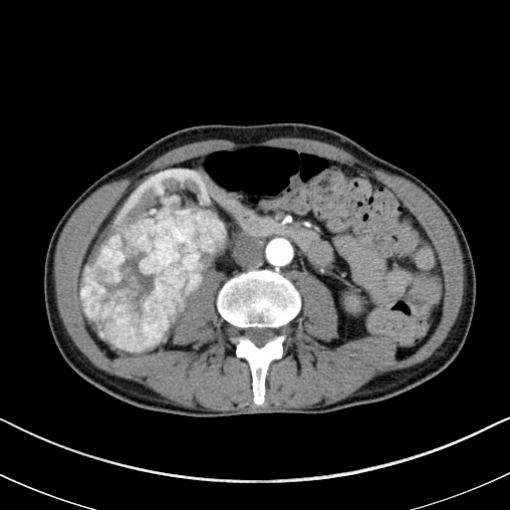
**CT scan showing large tumor of the right kidney**.

**Figure 2:**
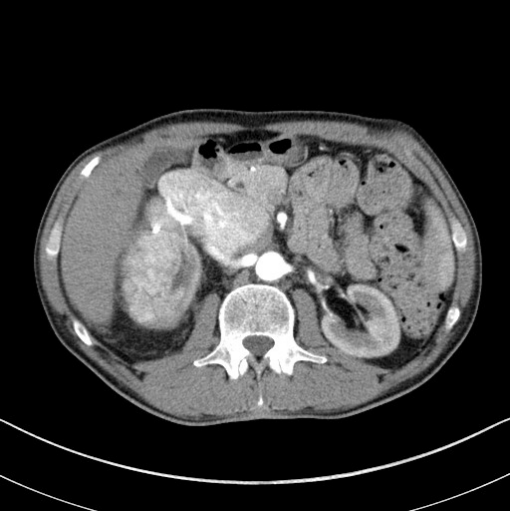
**CT scan showing involvement of infradiaphragmatic juxtahepatic part of inferior vena cava**.

**Figure 3:**
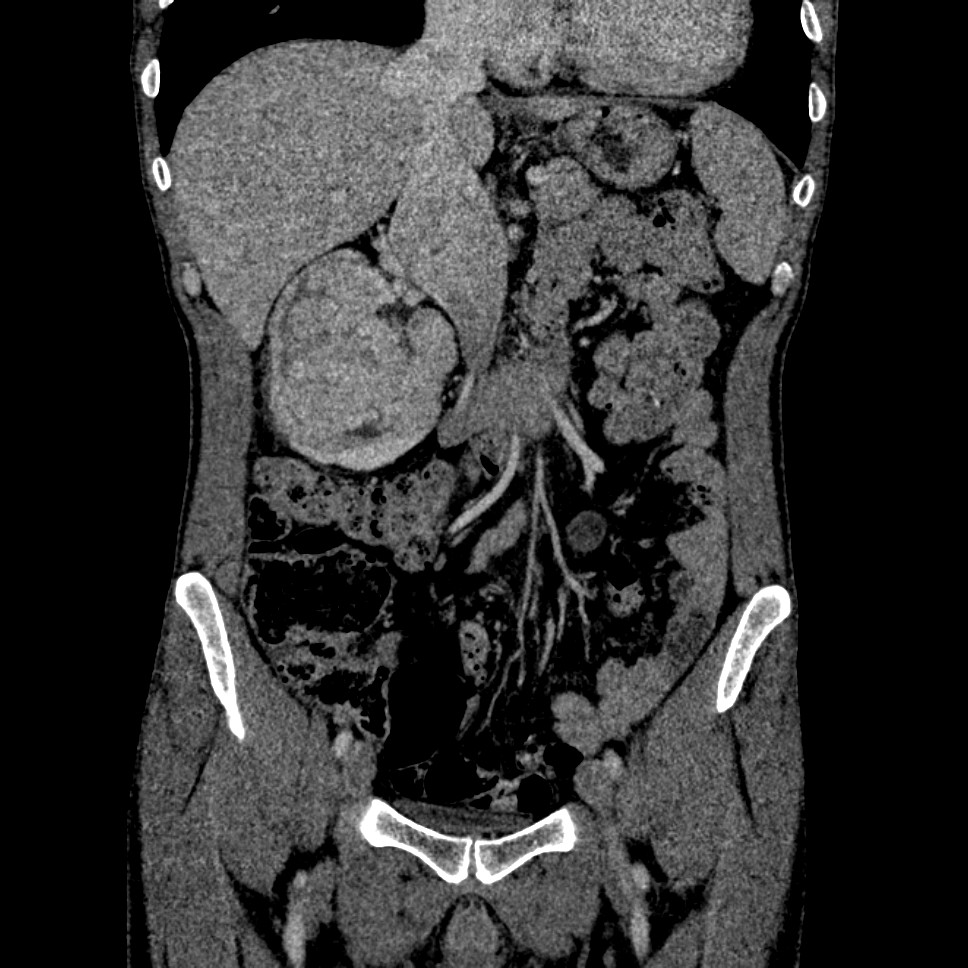
**CT reconstruction of the abdomen showing the size of the renal mass and the thrombus**.

On physical examination right lower abdomen mass was found and CT scan revealed large (12 cm × 11 cm × 7 cm) right kidney tumor with extension to infradiaphragmatic juxtahepatic part of inferior vena cava (caval thrombus 9 cm × 5 cm) (See Figures1,2,3). Several options of treatment were considered but during discussion with cardiac surgeons and urologists one stage operation was decided and carefully planed.

In preoperative period patient received hypotensive drugs (furosemide 0,04 g, spironolactone 0,025 g), beta-blocker (metoprolol 0,05 g), antyarrhytmic drug (amiodarone 0,2 g), anticoagulant (enoxaparin 0,06 g), potassium and magnesium.

Cardiac part of operation was performed first. Chest was open through median sternotomy and cardiopulmonary bypass (CPB) was established by cannulation of both venae cavae and ascending aorta. After clumping the aorta heart was stopped by cold blood cardioplegia, and both valves were repaired - dilated mitral annulus with C-G Future Band (Medtronic Inc.USA) and tricuspid annulus with De Vega plasty. After aortic clump was removed heart rhytm was restored with DC shock. CPB was discontinued without problems, patient was decannulated, heparin reversed with protamine. Transoesophageal echocardiogram confirmed good result of valves repair. The extracorporeal circulation time was 72 minutes, the aorta was clumped for 49 minutes.

**Figure 4:**
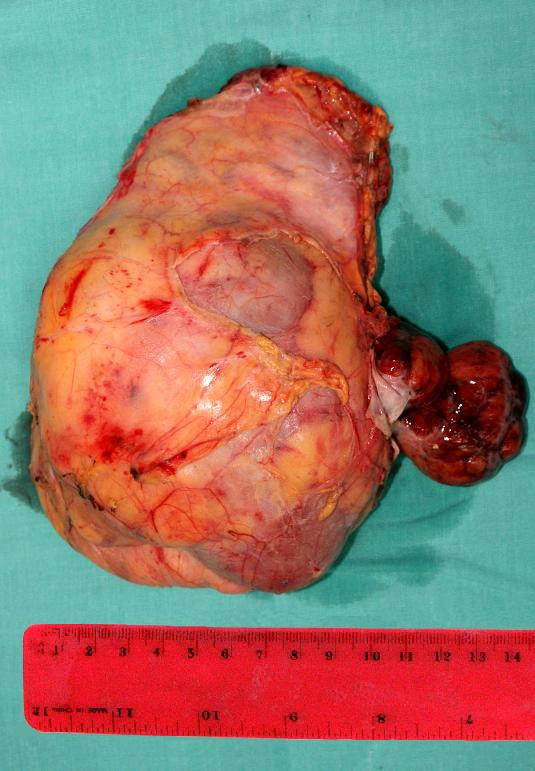
**Polymorphic appearance of renal cell carcinoma of size 12 × 11 × 7 cm**.

The second part of the operation was carried out just after the patient was hemodynamically stable. Urologists performed right radical nephrectomy through laparotomy. Accurate localization of the thrombus was assessed intraoperatively and a decision not to use cardiopulmonary bypass for thrombectomy was made. The kidney, the adrenal gland and the thrombus were removed intact (Figure 4).

**Figure 5:**
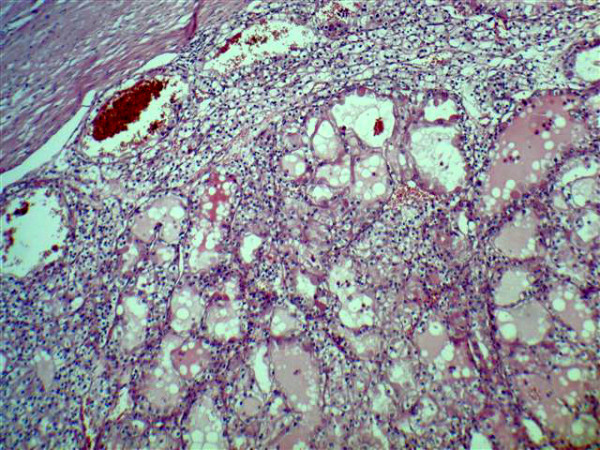
**Pathological findings of renal cell carcinoma**.

The operation took 4 hours 25 minutes. Blood loss was 600 ml. 5 units of fresh frozen plasma (5 × 220 ml), 2 units of red blood cells (2 × 500 ml) and 1 unit of platelets were administered. There were no complications. Macroscopic evaluation of the specimen showed 10 × 10 × 9 cm renal mass and 6 × 3 × 4 cm neoplasmatic thrombus. Microscopic examination revealed clear cell carcinoma of the kidney at the stage G1 pT3bN0M0, not infiltrating renal capsule (Figure5). 10 lymphatic nodules were negative.

In postoperative echocardiography there were no signs of mitral either tricuspid incompetence or pericardial effusion. Electrocardiogram demonstrated regular sinus rhythm. In peri- and postoperative period patient received antibiotic prophylaxis (ceftriaxone 2,0 g) , hypotensive drugs (furosemide 0,04 g, enalapril 0,05 g), beta-blocker (metoprolol 0,05 g), antyarrhytmic (amiodarone 0,2 g), anticoagulant (enoxaparin 0,06 g), acetylsalicylic acid (0,075 g), omeprazole (0,02 g), potassium and magnesium. 6 days after surgery oral anticoagulant therapy was started with acenocoumarol (0,002 g).

9 days after the operation the patient was transferred from cardiosurgical department to urologic centre. 4 days later he was discharged in good condition. 12-month follow-up showed that the patient remains without any complaints. Computed tomography demonstrated no signs of reccurrence.

## Conclusions

To the best of our knowledge, this is the first reported case of patient, who underwent one-stage mitral valvuloplasty, tricuspid valvuloplasty and radical nephrectomy with inferior caval vein thrombecotmy. Coexistence of combined valvular heart disease with severe heart failure and renal cell carcinoma infiltrating renal and inferior caval vein rendered this operation as very high risk procedure. However, the strategy of two separate operations was contraindicated irrespective of the order of interventions. Cardiac operation in patient with virtually total obstruction of inferior caval vein could not make an expected profit and additionally could be significantly unfavourable due to the delay of oncological treatment. On the other hand the risk of urologic operation in patient with so advanced circulatory insufficiency would be extremely high.

Till now there has been 4 reports on simultaneous cardiac procedure and nephrectomy enrolling in total 9 cases [6-9]. Among them there are no reports on such complicated operations neither on one-stage tricuspid valve nor kidney operations (Table 1).

**Table 1:** Data from the literature concerning one-stage cardiac operation and nephrectomy

Author	Study dates	Number of patients	Cardiac procedure	Urologic procedure	Operative mortality	Complications	Mean follow-up time	Follow-up results
Franke [6]	2000	1	CABG	nephrectomy and IVC thrombectomy	0%	No	9 months	excellent status, no signs of recurrence

Litmathe [7]	1989-2000	6	4 CABG, 2 aortic valvuloplasty	5 radical nephrectomy, 1 partial nephrectomy	0%	No	72 months	4 alive, 1 ischemic symptoms

Marino [8]	2008	1	AVR	radical nephrectomy	0%	No	0	No

Dedeilias [9]	2008	1	CABG	radical nephrectomy	0%	No	17 months	excellent status, no signs of recurrence

The aim of this report was to prove the possibility of simultaneous difficult cardiac and urologic operation. The most important point of our report concerns the fact that the oncologic treatment was not delayed despite severe heart disease. There is also an advantage in avoiding second operation and hence anesthesia. Essential disadvantages, which have to be considered are as follows: increased probability of bleeding due to heparinization, operation time, its complexity and risk of patient's death.

One-stage cardiac and uro-oncologic operation can be a safe and beneficial procedure, if performed in selected patients by experienced cardiosurgical and urological teams. There is a need of greater number of patients and long term follow-up to establish final conclusions.

## Consent

Written informed consent was obtained from the patient for publication of this case report and any accompanying images. A copy of the written consent is available for review by the Editor-in-Chief of this journal.

## Abbreviations

AVR: aortic valve replacement; CABG: coronary artery bypass grafting; CT: computed tomography; DC shock: direct current shock; EF: ejection fraction; NYHA: New York Heart Association; PASP: pulmonary arterial systolic pressure.

## Competing interests

The authors declare that they have no competing interests.

## Authors' contributions

AAA made substantial contributions to the conception and design of management and report, assisted in the urological part of the operation, analyzed and interpreted all data, and has been involved in drafting the manuscript; SP made substantial contributions to the acquisition of data, analysis and interpretation of data, assisted in the urological part of the operation, and has been involved in drafting the manuscript; ABi made substantial contributions to conception and design, performed the cardiac part of the operation, and has been involved in revising critically the manuscript for important intellectual content; LZ made substantial contributions to acquisition of data and helped in drafting the final version of English text; ABo made substantial contributions to conception and design, performed the urological part of the operation, has been involved in revising critically the manuscript for important intellectual content, and has given final approval of the version to be published. All authors read and approved the final manuscript.
